# Epidemiology, treatment outcome and resistance profile of pulmonary tuberculosis cases at the Niamey national anti-tuberculosis center in Niger: a retrospective study

**DOI:** 10.11604/pamj.2024.47.214.38442

**Published:** 2024-04-29

**Authors:** Mamane Djika, Charles Hornel Koudokpon, Victorien Tamègnon Dougnon, N'dira Sanoussi, Soumana Alphazazi, Boubacar Ballé, Hassane Daouda, Phénix Assogba, Honoré Bankole, Clément Agbangla

**Affiliations:** 1 National Anti-Tuberculosis Center, National Tuberculosis Control Program, Niamey, Niger,; 2 Laboratory of Molecular Genetics and Genome analysis, Faculty of Science and Technology, University of Abomey-Calavi, UAC, Cotonou, Benin,; 3 Research Unity of Applied Microbiology and Pharmacology of Natural Substances, Research Laboratory in Applied Biology, Polytechnic School of Abomey-Calavi, University of Abomey-Calavi, Cotonou, Benin,; 4 Laboratoire de Référence des Mycobactéries, Centre National Hospitalier Universitaire de Pneumo-Phtisiologie de Cotonou, National Tuberculosis Programme, Cotonou, Benin

**Keywords:** Tuberculosis, diagnosis, treatment, resistance

## Abstract

**Introduction:**

tuberculosis remains a major public health problem, with continuing high levels of prevalence, and mortality. In Niger, the incidence of tuberculosis remains high. This study aims to investigate the epidemiology of pulmonary tuberculosis at the National Anti-Tuberculosis Center of Niamey in Niger.

**Methods:**

this study used a quantitative approach with a retrospective and descriptive design. Data were obtained from positive pulmonary tuberculosis cases detected by microscopy on Ziehl-Neelsen stained sputum at the National Anti-Tuberculosis Center (NATC) in Niamey, Niger covered the period between June 2017 and January 2020. 955 pulmonary TB patients were recorded whose diagnosis was based either on clinical-radiological arguments (thus negative microscopy) or positive microscopy. This form was used to collect data recorded in the clinical case registers, registers, and Excel files of the GeneXpert platform of the NATC laboratory.

**Results:**

eighty-nine-point eleven percent (89.11%) of the patients were microscopy-positive. Among the study population, men were the most affected by tuberculosis with 80.03%. The 25-34 age group, representing 23.77%, was the most affected. 6.93% of patients were co-infected with tuberculosis and HIV. All patients were put on treatment, with a therapeutic success rate of 72.38% and a therapeutic failure rate of 10.95%. Among the cases of therapeutic failure, 80.90% had Mycobacterium tuberculosis complex detected and 27.14% were resistant to Rifampicin.

**Conclusion:**

Niger continues to have a tuberculosis epidemic which requires monitoring. Improving the diagnostic system for more effective management of the disease is important for appropriate diagnosis and treatment.

## Introduction

Tuberculosis is a disease as old as humankind that, despite the discovery of effective antibiotics for its treatment, continues to kill thousands of people worldwide, especially in resource-limited countries [1]. Globally, TB is one of the top 10 causes of death and the leading cause of death from a single infectious agent ahead of HIV/AIDS. Globally, an estimated 10 million people (range: 8.9-11.0 million) had TB in 2021 [2]. In 2019, the number of TB deaths was estimated to be 1.2 million (range: 1.1-1.3 million) among HIV-negative individuals, with an additional 208,000 deaths (range: 177,000-242,000) among HIV-positive individuals [2]. The incidence of TB varies from country to country. The African region continues to bear a large share of the global TB burden, accounting for 25% of the 9.6 million incident TB cases worldwide [2]. In Africa, the highest rate is observed in Guinea-Bissau at 361 per 100,000 population, and the lowest in Togo at 36 per 100,000. In 2018, the incidence of TB in Africa was 275 (range: 238-314) per 100,000 population, compared with 10.7 per 100,000, for example, in the European Union. In addition, of the 30 countries most affected by TB worldwide, three are in West Africa, three are in Central Africa, six are in East Africa, and four are in southern African countries [3]. Although the TB epidemic has declined modestly over the past decade, the incidence remains very high in low-and middle-income countries, most notably in Africa. Moreover, the disease burden is expected to persist or even increase in Africa for many years due to the lack of an effective vaccine and short treatment regimens.

The health goals of the recently adopted Sustainable Development Goals call for the eradication of the TB epidemic by 2030. WHO has gone even further and set a target of a 95% reduction in deaths and a 90% reduction in TB incidence by 2035, which is consistent with current levels in countries with low TB incidence [4]. Achieving these goals requires monitoring the global TB situation and measuring progress in TB care, control, and financing. It also involves stimulating the generation, translation, and dissemination of valuable knowledge [4]. A better understanding of TB epidemiology, particularly in Africa, is essential to enable more targeted interventions to overcome transmission chains and mitigate or eliminate current disease trends [5]. The recent development of modern molecular epidemiology techniques, however, provides a better understanding of the dynamics of TB transmission through the identification and assessment of the molecular characteristics of circulating mycobacterial strains, resistance patterns, and factors associated with the disease [6].

Niger is one of the countries most affected by TB with an incidence rate of 87 cases per 100,000 population with a low target coverage rate of 57% in 2018 [7]. The treatment success rate is 82% for the 2017 cohort in the face of an estimated 2.4% of new cases with MDR/XDR-TB compared to the average for West African countries, which ranges from 0.9% in Senegal to 4.6% in Côte d'Ivoire [7]. Nevertheless, much data on the evolution of pulmonary tuberculosis in recent years is still unavailable, thus blocking many actions aimed at achieving the objectives for the eradication of pulmonary tuberculosis. It is to overcome this lack that the present study was initiated and aimed at investigating the epidemiology pulmonary tuberculosis epidemiology cases in Niger.

## Methods

**Study setting and design:** this study used a quantitative approach with a retrospective and descriptive design. Data were obtained from positive pulmonary tuberculosis cases detected by microscopy on Ziehl-Neelsen stained sputum at the National Anti-Tuberculosis Center (NATC) in Niamey, Niger. It is the reference center for tuberculosis in Niger, where patients received in the tuberculosis centers of Niamey and its surroundings are referred and screened for microscopically positive TB. 955 pulmonary TB patients were recorded whose diagnosis was based either on clinical-radiological arguments (thus negative microscopy) or positive microscopy. The study focused on patients with positive microscopy regarding the WHO concept of “bacteriologically confirmed” [8]. The study covered the period between June 2017 and January 2020.

**Sampling size:** no sampling method was used in this study. In order to be comprehensive, we included data from all patients tested during the study period for tubercle bacilli on Ziehl-Neelsen-stained sputum microscopy.

**Study variables:** the dependent or outcome variable in this study was the positivity of new cases to Ziehl-Neelsen stained sputum microscopy. Independent variables included: age, sex, diagnostic microscopy results, follow-up monitoring results, treatment outcome, HIV status, and for some patients, Xpert MTB/RIF test results.

**Data collection:** as data collection tools, we used a survey form designed for this study. This form was used to collect data recorded in the clinical case registers, registers, and Excel files of the GeneXpert platform of the NATC laboratory. For each patient, we collected the following data: age, sex, diagnostic microscopy results, follow-up monitoring results, treatment outcome, HIV status, and for some patients the results of the Xpert MTB/RIF test. At NATC, every patient who tests positive for TB is counseled for HIV testing. After obtaining the patient's consent, HIV testing was performed using the rapid immunochromatography test on the patient's blood with the Determined Alere HIV 1/2. Positive results are discriminated by the BiSpot HIV 1/2 Immuno Comb test [9,10].

**Statistical analysis:** the data extraction was conducted using Microsoft Excel 2019. The formal data analysis was conducted using EpiData v1.1.2.62 and Epi Info v7 software. The Pearson Chi-squared test was used for comparisons and analyses were conducted with a P-value of 0.05 considered statistically significant.

**Ethical consideration:** this study was approved by the National Health Research Ethics Committee by Deliberation N°001/2019/CNERS of 14^th^ March 2019. The forms used to extract data for this study were anonymous and confidential.

## Results

**Socio-demographic characteristics:** a total of 955 new cases of suspected pulmonary tuberculosis were included, of which 851 had a positive microscopy (89.11%) versus 104 with a negative microscopy (10.89%). The majority of cases were Males representing 80% (n=683), and the percentage of Females was 20% (n=168). The Age of patients was raging from 0.8 to 90 years. The Median age was 35 with the interquartile range being (25,44). The HIV status and sex of patients are shown in Table 1. It can be noted that among these positive cases detected through TB microscopy screening, only 6,32% (n=59 tested positive for HIV).

**Table 1:** distribution of MPT by HIV status and sex

SEX	MTP+	MTP-	Total	P value	OR
HIV +	59 (6,32%)	16 (1,71%)	75 (8,03%)	0.0001	0,32
HIV -	790 (84,58%)	69 (7,39%)	859 (91,97%)		
TOTAL	849 (90,90%)	85 (9,10%)	934 (100%)		
HIV ND	21				
**SEX**	**MPT+**	**MTP-**	**Total**	**P value**	**OR**
Male	683 (71,52%)	62 (6,49%)	745 (78,01%)	0.0001	0,45
Female	168 (17,59%)	42 (4,40%)	210 (21,99%)		
TOTAL	851 (89,11%)	104 (10,89%)	955 (100%)		

**Epidemiology of microscopy-positive pulmonary tuberculosis:** Table 2 shows the age group distribution of microscopy-positive pulmonary tuberculosis. The highest frequencies were noted in the age group of 20 to 30 years. All patients detected positive by microscopy were systematically put on treatment. However, controls were carried out after two months (M2), five months (M5), and six months (M6) of treatment. Thus, at the end of the control carried out in the second month of treatment for the 851 positive patients, it was found that 36.07% were positive, At M5 and M6, the respective positivity of the smears was noted at 13.06% and 4.95%. The Ziehl-Neelsen stained sputum microscopy results are shown in Table 3.

**Table 2:** age distribution of new pulmonary TB patients

Age	Positive	Negative	Total	P value	OR
**[0-5]**	8 (0,84%)	21 (2,20%)	29 (3,04%)	0,0001	1,30
**[5-10]**	2 (0,21%)	14 (1,47%)	16 (1,68%)		
**[10-15]**	11 (1,15%)	7 (0,73%)	18(1,88%)		
**[15-20]**	52 (5,45%)	7 (0,73%)	59 (6,18%)		
**[20-25]**	125 (13,09%)	13 (1,36%)	138 (14,45%)		
**[25-30]**	123 (12,88%)	6 (0,63%)	129 (13,51%)		
**[30-35]**	104 (10,89%)	7 (0,73 %)	111 (11,62%)		
**[35-40]**	114 (11,94%)	4 (0,42%)	118 (12,36%)		
**[40-45]**	99 (10,37%)	4 (0,42%)	103 (10,79%)		
**[45-50]**	83 (8,69%)	6 (0,63%)	89 (9,32%)		
**[50-55]**	24 (2,51%)	4 (0,42%)	28 (2,93%)		
**[55-60]**	33 (3,46%)	4 (0,42%)	37 (3,87%)		
**[60-65]**	22 (2,30%)	3 (0,31)	25 (2,62%)		
**[65-70]**	16 (1,68%)	1 (0,10%)	17 (1,78%)		
**[70 et + ]**	35 (3,66%)	3 (0,31%)	38 (3,98%)		
**Total**	851 (89,11%)	104 (10,89%)	955 (100%)		

**Table 3:** Ziehl-Neelsen stained sputum microscopy results

Microscopic tests	Positive	Negative	Total
Prevalence	89,11%	10,89%	100%
Month 2 control	36,07%	63,93%	100%
Month 5 control	13,6%	86,4%	100%
Month 6 control	4,95%	95,05%	100%

**Figure 1:**
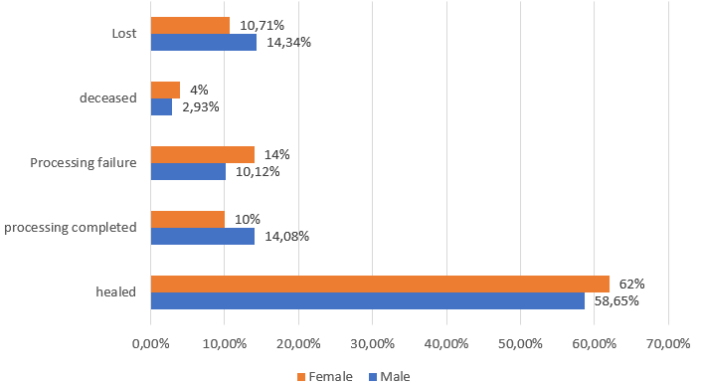
treatment outcomes of patients started on treatment and followed with NATC

**Treatment outcome:** Figure 1 shows the treatment outcomes of patients who started treatment and were followed by NATC. Of the 851 cases started on treatment and followed up by NATC, from an overall point of view, the results of the treatment showed that the treatment success was 72.38% (616/851), the treatment failure was 10.93%, death cases 3.06% (26/851), and 116 patients or 13.63% were lost to follow-up. In men, treatment success was 72.62% (496/683). The frequency of end of treatment was lower in women with 10% against 14.08% in men. Treatment failure was more common among women (14%) than among men where it was 10.12%. The mortality rate for men was 2.93% compared to 4% for women.

**Figure 2:**
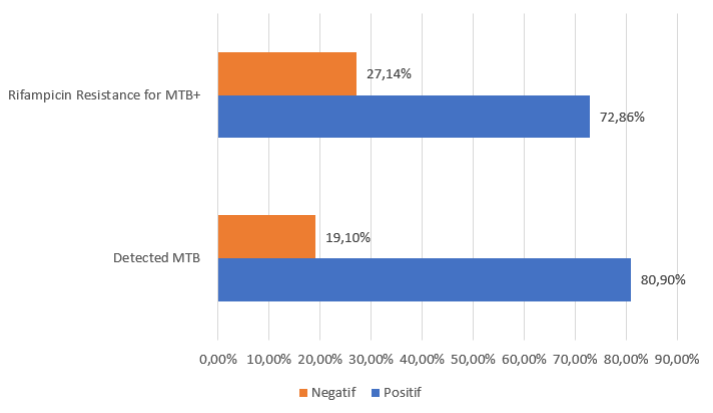
GeneXpert result for processing failure

**Resistance to rifampicin:** TPM-positive patients with a treatment failure outcome were tested with Xpert MTB/RIF molecular testing for Mycobacterium tuberculosis complex (MTB) and rifampicin resistance (RIF). It was noted that the Xpert test was not performed in 4 of the 93 patients with treatment failure. Thus, in 72/89, ie 80.9% of them, the Mycobacterium tuberculosis complex was indeed detected. 56/72 or 77.78% of patients in whom MTB was detected were male. In 19.1% (17/89) of patients, MTB was not detected and 9/17 or 52.94% were male (Figure 2). Patients with Mycobacterium tuberculosis complex (MTB) were tested for rifampicin resistance. 72.86% of patients presented a sensitive profile against 27.14% who were resistant to rifampicin. A single positive MPT in a situation of treatment failure had a positive HIV serology of 1/19, or 5.3%. Three types of mutations have been identified: type B, type D, and type E. A type A+D double mutation has been identified in one patient. The most common mutation is type B (6/16).

## Discussion

The objective of this study was to produce scientific data on the evolution of pulmonary tuberculosis epidemiology from 2017 to 2020 in Niamey, Niger. Globally, according to the most reliable estimates, 10.0 million people contracted TB in 2018. In Niger, the incidence of TB was 87 cases per 100,000 population. Pulmonary forms observed in incident cases consisting of new cases and relapses accounted for 87%, of which 92% were bacteriologically confirmed [11]. Our study reviews the epidemiological and evolutionary profile of new pulmonary TB cases in NATC from June 2017 to January 2020.

During this period, 955 new TB patients were received at the NATC. Men represented 78.01% against 21.99% for women, i.e., a sex ratio of 3.54. This male predominance has been reported by other authors [12,13]. In daily life, women stay at home much more than men. This state of affairs constitutes, in itself, a limitation to the exposure to tubercle bacilli in public transport public places where the probability of contamination would be higher. In Brazil, in the municipality of Bethlehem, the number of cases is very similar among men and women, but it is shown that the male gender is more affected by the disease [14]. The clear predominance of men (sex ratio of 1.4), which is found in many developing countries, is partly explained by the different lifestyles between the sexes and the fact that women are generally less available than men to consult and look after their health. The mean age of the patients was 34.88 years, the modal age was 35 years, and the median was 33 years with extremes of 0.3 years and 90 years. The most affected age group was 25-34 years with 25.13% (240/955). In Congo (DRC), the most affected age groups were 21-30 years (28.24%) and 31-40 years (26.55%) [15].

Of the 955 new TB patients admitted to NATC from June 2017 to January 2020, the diagnosis was based on bacteriology and/or clinical or radiological arguments. In Niger, the TB diagnosis algorithm includes microscopy as the first line for bacteriological confirmation (except for some special cases: children aged 0-14 years, PLHIV, elderly, and in case of suspected MDR-TB) and then as a follow-up examination. Smear microscopy, also known as bacilloscopy, remains the cornerstone of the diagnosis of pulmonary TB in adults because it identifies the most potent sources of disease transmission. A positive bacilloscopy is a marker of the infectiousness of this disease. It can be performed rapidly and has high specificity in high-prevalence countries [16]. 851/955 patients (89.11%) had bacteriologically confirmed tuberculosis through microscopy; thus, they are microscopy-positive pulmonary tuberculosis cases (MPT positive). In a recent study in Maradi, Central Niger, the rate of bacteriology (microscopy) confirmed TB was 64.7% [17]. This difference in rate suggests that Maradi bacteriological confirmation is lower than at NATC, which is still the national reference center for tuberculosis. This fact predisposes it to receive, as a reference, complicated cases that would be more bacilliferous. The rate of 89.11% of positive PMT is higher than that found in Madagascar where it is nearly 80% in patients with pulmonary forms of tuberculosis [15] and in Mayotte where it is 44.7% of cases with pulmonary form [18]. The age group of more than 25 years was the most affected with 76.7% of positive PMT cases against 23.3% for the 0-24 age group (Chi^2^ Squared: 222.4125 for ddl = 4). The occurrence of tuberculosis in this population is related to age. In general, in sub-Saharan Africa, tuberculosis affects the 20-45 age group with a clear male predominance. Indeed, young adults, especially males, are the most economically productive and are found in various sectors of activity [19]. However, tuberculosis affecting these able-bodied individuals could have an economic impact. Among the MPTs who were positive, 6.93% had positive HIV serology. This rate of TB-HIV co-infection is lower than the rate found in Maradi in 2019, which was 13.6% [17]. On the other hand, this coinfection rate is approximately equal to that found in *Saint-Louis du Sénégal*, which is 6.3% [11]. The rate observed in our study is higher than the TB-HIV co-infection rate of 1% in Mayotte, but it should be noted that in our study all positive PMTs were tested for HIV, whereas in Mayotte 63.5% of the cases have an HIV status [18]. Seropositivity is more frequent among women 14.28% than men and 5.12% IC 95 [1.68%-5.36%]. Seropositivity among TB patients is 6.93% [5.45-7.22%], apparently low compared to that of women. Moreover, seropositivity seems to be associated with sex (Odds Ratio = 3.09; k^2^ =17.51, p<0.00003) and it is more likely that a female TB patient is HIV positive than a male.

The NATC is the national reference center for the management of tuberculosis and respiratory diseases. It receives patients from various facilities in Niger. Referred patients receive specialized consultations and laboratory tests. For many referred patients, once the diagnosis of tuberculosis has been made, they return to the referring center for treatment and follow-up. Only patients who have made the first consultation at this center are registered for treatment and follow-up by the NATC. From June 2017 to January 2020, 851 new TPM-positive TB patients were seen and put on treatment and follow-up at NATC. The treatment regimen is the RHZE (Rifampicin, Isoniazid, Pyrazinamide, Ethambutol) combination. It is a six-month regimen including two months of intensive phase (2RHZE) and four months for the continuation phase (4RH). Follow-up microscopy showed a change in the conversion rate of positive smears from 63.93% at the second month (M2) to 86.4% at the fifth month (M5) and 95.05% at the last end-of-treatment check (M6). Microscopy is a follow-up examination that allows a good assessment of the effectiveness of the treatment. Taking into account this variation in the overall positive smear conversion, we can say that the NATC patient management strategy is effective. This strategy combines the free administration of anti-tuberculosis drugs and awareness-raising activities. However, there is a wide variation in the number of patients who did not undergo this follow-up examination. Indeed, from 8.5% of TPM-positive patients who did not undergo M2 follow-up bacilloscopy, we observed 16.0%, i.e., double the number of patients who did not undergo the M5 control microscopy, and finally, 35.8% did not undergo the M6 control. These patients who did not respond to the control microscopy are essentially patients who were lost to follow-up, those who died, and those who deliberately decided not to do this examination. This may be due to incomplete adherence to the advice given by the nursing staff and social workers. Indeed, the NATC has a social service that explains and advises on what to do with any patient put on treatment. This lack of adherence could be linked to ignorance of the importance of follow-up examinations, which are carried out very early in the morning, while the medication is given at a different time of day. Patients who feel that their health is improving prefer to come to the NATC at a time when they are sure to have the medication.

Among the M2 negative patients, 17 were positive at the M5 control, i.e., 3.72%. Among these 17 patients, 9 had therapeutic failure and therefore benefited from the Xpert test. Five (5) of them showed a sensitivity profile to rifampicin while four (4) were resistant. Considering that the treatment is a standard, six-month regimen with the 2RHZE4RH combination, the availability of these anti-tuberculosis drugs, and their free distribution, one could only incriminate factors related to the drugs and or the patients. Indeed, for some patients, the lack of transportation to the NATC means that they find themselves in a situation of drug shortage; on the other hand, for other patients, it is the side effects that cause noncompliance with the regularity of taking the drugs. All these factors can have an impact on the reversion of their sputum smears, either individually or in combination. Of the 851 microscopically confirmed new cases, 9 patients were positive for all 3 controls M2, M5, M6 (1.1%). The fact that patients are still detected positive at the next control could be explained by the fact that the patient does not adhere properly to the treatment, or that he/she harbours two strains, one of which responds to the treatment and the second one which is resistant would then be favoured by selection pressure, or simply that the strain was resistant from the start. Microscopy is an examination that detects BAARs without discriminating between the tuberculosis complexes and atypical mycobacteria. Even if it is a tuberculosis complex, bacilloscopy does not give the antibiotypic profile. The treatment success rate was 72.55%, which is lower than the national rate of 82%, and found in Mayotte where the treatment success rate is 79.8% [20]. One of the indicators for performance evaluation of National TB Programs is the treatment success rate.

For WHO, the recommended cure rate for resource-limited countries is 85% of newly detected TB patients. The overall frequency of completed treatment was 13.19% (112/851). This frequency was lower in women (10%) than in men (14.08%). Treatment failure was 10.95% and this was more frequent in women (14%) than in men where it was 10.12%. The death rate was 3.06%, whereas in Mayotte it was 6.4%, i.e., twice as high. The death rate for men was 2.93% compared to 4% for women. This higher death rate among women could be explained by the fact that they consult the health services much later than men; even if they are diagnosed with tuberculosis, they are less regular in their attendance at the health service and therefore subject to complications, including death. This situation could be explained by the socio-cultural context which makes women less independent in their movements than men. 116 patients or 13.63%, 95% CI [13.60-13.65] were lost to follow-up, whereas WHO standards call for a rate of 5% or less in a cohort. The LOS rate found in Maradi is 7.4% [17]. The VDP rate found in our study is still much lower than the rate found in Mayotte which is 41.1%. The VDP rate in our study is also lower than the dropout rate in the Brazzaville health region in Congo, which is 22.5% [18]. At 14.34% 95% CI [14.32-14.37] in men, this rate is significantly higher than in women, where it is 10.71% 95% CI [10.68-10.73].

The high rate of VDP observed in our study could be explained by factors related to the patient, such as financial accessibility; the absence of a fixed address for patients who are not from Niamey. We can also mention factors related to the health professional: poor reception, unavailability of health personnel for patients who come at certain times of the day. The phenomenon of POS could also be due to factors related to the drugs, namely, the occurrence of undesirable effects; the long duration of treatment which discourages patients from respecting the regularity of taking the drugs. Factors related to the organization of health services: patient circuit (several comings and goings) and the lack of permanence after working hours negatively impact the respect of appointments and after a certain time patients end up being POS. The rate of POS in various African and Asian countries varies from 10% to 23% depending on the study [20]. This phenomenon was observed within all age groups with a peak of 32.14% in the 25-34-year-old age group. Among the POS, 59 of them had received follow-up control at the intensive phase, of which 24 or 40.68% had a positive smear. 17 VDPs were included in the follow-up check-up in the continuation phase and 7 (41.18%) of them were smeared positive. Being lost to follow-up during treatment of active TB can have potentially serious consequences for the patient, as well as for public health, given the risk of spreading the disease.

TPM-positive patients with failed treatment outcome 89/93 (95.7%) were subjected to the Xpert MTB/RIF molecular test for Mycobacterium tuberculosis complex (MTB). In 19.1% (17/89) of patients, MTB was not detected. One of the limitations of microscopy is that it cannot discriminate between TB complex and non-tuberculosis complex BAARs. Given that the Niger algorithm uses microscopy as the first test, then these patients with mycobacteria will always be managed as TB patients. It is the positivity of their smears at M5 or completely at the end of the treatment that the treatment failure will be noted. In 72/89 or 80.9% of patients in a treatment failure situation, MTB was indeed detected. These patients were tested for rifampicin resistance. Of these, 72.86% (51/70) had a sensitive profile and 27.14% (19/70) had rifampicin resistance. One of the limitations of the current algorithm in Niger is its inability to detect cases of rifampicin resistance at diagnosis and cases of mycobacteriosis. Patients in these two situations will be treated as rifampicin-susceptible TB patients. This situation leads to inappropriate use of drugs and the spread of resistant strains to the patient's environment. Resistance to rifampicin affected the age groups from 15 years to more than 45 years with a peak of 42.10% for the 15 to 24 years age group. Only one (1/19) or 5.3% of the MPT positives in a treatment failure situation had positive HIV serology. Among the 9 MPT+ who remained positive during the three controls, MTB was not detected by the Xpert test in 4 patients but was detected in 5/9 (55.56%); 4/5 of these patients were tested for rifampicin resistance, 1/4 of whom showed rifampicin resistance. This patient would have had primary resistance that should have been detected even before treatment was started if the Xpert test had been used in the first instance. This is one of the clear advantages of genotypic testing in the diagnosis of TB.

**Limitations:** our study considered the sentinel sites established throughout the country. However, it's important to note that our results do not encompass sites that are not integrated into the current surveillance system in Mali. Nevertheless, the number of non-integrated sites is relatively small compared to the sites included in our study. The selected sites are reasonably representative of Niger's healthcare situation. The biases inherent to the retrospective nature of the study and the extensive geographical scope (including data loss and other factors) were also minimized through supervisions during data collection. Despite these precautions, it's important to acknowledge that these biases cannot be entirely eliminated.

## Conclusion

This study of new pulmonary tuberculosis patients at the NATC in Niamey revealed that the male sex is still the most affected and that these patients are mostly young adults. TB/HIV co-infection was noted. Despite an appreciable rate of therapeutic success, it remains below the WHO standard. Our study has shown that the number of patients lost to follow-up is a significant fraction of the total number of patients, which will require the implementation of innovative strategies to not only understand this phenomenon but also to reduce the number of patients lost to follow-up. Microscopy being, in Niger, the first intention examination and basis for treatment, could be among the factors of the worrying rate of treatment failure. Indeed, among the microscopy-positive patients put on treatment with the sensitive tuberculosis regimen, some are suspected of having mycobacteriosis by the Xpert test. In addition, the Xpert MTB/RIF test detected that 27.14% of TB patients were carriers of rifampicin-resistant strains, in contrast to microscopy. These patients could have been put on the RR-TB (rifampicin-resistant TB) treatment regimen earlier than the 5-6 months it took to perform the Xpert test at the time of treatment failure; this would also avoid the transmission and spread of rifampicin-resistant strains.

### What is known about this topic

- *Niger was known as one of the country's most severely affected by tuberculosis*.

### What this study adds

- *Expanded tuberculosis epidemiology assessment: our study surpasses previous data on tuberculosis prevalence in Niger by adopting a more comprehensive approach; Unlike previous methods that primarily relied on microscopy, our study delves deeper, providing insights into crucial aspects such as resistance to first-line antibiotics like rifampicin;*
- *Correlation with HIV program implementation: collaborating with a new program in Niger allowed us to establish correlations between tuberculosis and HIV rates within the population of people living with HIV (PLHIV); this integration of data sheds light on previously overlooked connections and enhances our understanding of the epidemiological landscape;*
- *Innovative treatment approach and statistical analysis: introducing a novel treatment approach, as detailed in our study, contributes to the field by offering previously undocumented strategies; furthermore, our utilization of a sophisticated statistical approach not only strengthens the robustness of our findings but also provides a wealth of additional information, enriching the comprehensiveness of our study*.

## References

[1] Kone B, Somboro AM, Holl JL, Baya B, Togo AA, Sarro YDS (2020). Exploring the usefulness of molecular epidemiology of tuberculosis in Africa: A systematic review. Int J Mol Epidemiol Genet.

[2] Organisation Mondiale de la Santé (2020). Rapport sur la tuberculose dans le monde, 2020. Licence: CC BY-NC-SA 3.0 IGO.

[3] Yimer SA, Birhanu AG, Kalayou S, Riaz T, Zegeye ED, Beyene GT (2017). Comparative proteomic analysis of Mycobacterium tuberculosis lineage 7 and lineage 4 strains reveals differentially abundant proteins linked to slow growth and virulence. Front Microbiol.

[4] Kashongwe MI, Mbulula L, Umba P, Lepira FB, Kaswa M, Kashongwe ZM (2017). Factors associated with mortality among multidrug resistant tuberculosis MDR/RR-TB patients in Democratic Republic of Congo. Journal of Tuberculosis Research.

[5] Peter M (2009). Tuberculosis: a new vision for the 21^st^ century. Kekkaku.

[6] Kamerbeek J, Schouls LEO, Kolk A, Van Agterveld M, Van Soolingen D, Kuijper S Bunschoten A (1997). Simultaneous detection and strain differentiation of Mycobacterium tuberculosis for diagnosis and epidemiology. J Clin Microbiol.

[7] Segbedji KAR, Djadou KE, Tchagbele OB, Kpegouni M, Kama LB, Azoumah KD (2016). Tuberculose de l´enfant au, Togo: aspects épidémiologiques, diagnostiques, thérapeutiques et évolutifs. Médecine et Santé Tropicales.

[8] Organisation Mondiale de la Santé (2016). Cadre pour la mise en œuvre de la «stratégie de l´OMS pour mettre fin à la tuberculose» dans la Région africaine au cours de la période 2016-2020: rapport du Secrétariat. No. AFR/RC66/10. Bureau régional de l'Afrique: OMS.

[9] Slim-Saidi L, Mehiri-Zeghal E, Ghariani A, Tritar F (2015). Nouvelles méthodes de diagnostic de la tuberculose. Rev Pneumol Clin.

[10] Truffot-Pernot C, Veziris N (2011). Les tests bactériologiques de la tuberculose maladie: standards et perspectives. Rev Mal Respir.

[11] Yaya I, Sissoko D, Guinan JC, Dosso M (2018). Épidémiologie de la tuberculose en Côte d´Ivoire: une revue systématique de la littérature/Epidemiology of tuberculosis in Côte d´Ivoire: a Systematic Review. Rev int sc méd.

[12] Ngama CK, Muteya MM, Lukusha YII, Kapend SM, Tshamba HM, Makinko PI (2014). Profil épidémiologique et clinique de la tuberculose dans la zone de santé de Lubumbashi (RD Congo). Pan Afr Med J.

[13] Niang S, Thiam KH, Mbaye FBR, Dieng A, Diop Dia A, Dia D G (2019). Profil épidémiologique, clinique et radiologique de la tuberculose pulmonaire A microscopie positive (TPM+) au centre hospitalier régional universitaire de Saint-Louis (CHRU SL). Mali Med.

[14] Homolka S, Post E, Oberhauser B, George AG, Westman L, Dafae F (2008). High genetic diversity among Mycobacterium tuberculosis complex strains from Sierra Leone. BMC Microbiol.

[15] Rakotondramarina D, Razafimalala F, Andrianaivo P, Rabeson D, Andriatsiva R, Andrianavalomahefa W (2000). Aspects épidémiologiques de la tuberculose dans le Moyen-ouest malgache. Bull Soc Pathol Exot.

[16] Van Deun A, Portaels F (1998). Limitations and requirements for quality control of sputum smear microscopy for acid-fast bacilli Planning and Practice. Int J Tuberc Lung Dis.

[17] Amadou MLH, Abdoulaye O, Amadou O, Biraïma A, Kadri S, Amoussa AAK (2019). Profil épidémiologique, clinique et évolutif des patients tuberculeux au Centre Hospitalier Régional (CHR) de Maradi, République du Niger. Pan Afr Med J.

[18] Woessner J, Receveur MC, Malvy D, Taytard A (2008). Épidémiologie de la tuberculose à, Mayotte. Bull Soc Pathol Exot.

[19] Kouassi B, Horo K, N´douba KA, Koffi N, Ngom A, Aka-Danguy E, Dosso M (2004). Profil épidémiologique et microbiologique des malades tuberculeux en situation d´échec ou de rechute à Abidjan. Bull Soc Pathol Exot.

[20] Charmillon A (2014). Tuberculose en Seine-Saint-Denis: déterminants de la perte de vue (PhD Thesis). Université de Lorraine.

